# Evaluation of Sleep Health in Children With Congenital Adrenal Hyperplasia Due to 21-Hydroxylase Deficiency

**DOI:** 10.1210/clinem/dgae836

**Published:** 2024-11-28

**Authors:** Laura Golob, Yesica Mercado-Munoz, Wenxi Liu, Anvita Singh, James S Hodges, Lianne Siegel, Helena Morero, Zan Gao, Angela Tipp, Stacey L Simon, Kyriakie Sarafoglou

**Affiliations:** Division of Pediatric Endocrinology, University of Minnesota Medical School, Minneapolis, MN 55454, USA; Division of Pediatric Endocrinology, University of Minnesota Medical School, Minneapolis, MN 55454, USA; Department of Physical Education, Shanghai Jiao Tong University, Shanghai 200240, China; Division of Pediatric Endocrinology, University of Minnesota Medical School, Minneapolis, MN 55454, USA; Division of Biostatistics and Health Data Science, University of Minnesota School of Public Health, Minneapolis, MN 55455, USA; Division of Biostatistics and Health Data Science, University of Minnesota School of Public Health, Minneapolis, MN 55455, USA; Division of Pediatric Pulmonary and Sleep Medicine, University of Minnesota Medical School, Minneapolis, MN 55454, USA; School of Kinesiology, University of Tennessee, Knoxville, TN 37996, USA; Neonatal Care, University of Minnesota Medical Center, Minneapolis, MN 55455, USA; Department of Pediatrics, University of Colorado Anschutz Medical Campus, Aurora, CO 80045, USA; Division of Pediatric Endocrinology, University of Minnesota Medical School, Minneapolis, MN 55454, USA; Department of Experimental and Clinical Pharmacology, University of Minnesota College of Pharmacy, Minneapolis, MN 55455, USA

**Keywords:** congenital adrenal hyperplasia, sleep, hypothalamic-pituitary-adrenal axis, cortisol, hydrocortisone

## Abstract

**Context:**

Literature on sleep health in children with congenital adrenal hyperplasia (CAH) is sparse despite the important role the hypothalamic-pituitary-adrenal axis plays in sleep onset, duration, and awakenings after sleep onset.

**Objective:**

To evaluate sleep health in children and adolescents with CAH as measured by wrist actigraphy and validated sleep questionnaires.

**Methods:**

Cross-sectional study at our multidisciplinary CAH clinic. Participants aged 3 to 18 years with classic CAH wore an ActiGraph GT3X+ accelerometer for 1 week. Children and parents completed sleep questionnaires, and the results were compared to published samples from the community and children with sleep disorders (clinical). Actigraphy sleep health measures were compared to consensus sleep duration recommendations and normative data in healthy children.

**Results:**

Forty-four participants (23 male) with CAH completed the study. Actigraphy found sleep duration in children with CAH was less than recommended guidelines with significantly worse sleep efficiency and increased wake after sleep onset (*P* < .05) compared to healthy children. After sleep onset, the average number of awakenings increased from 1.67 per hour during the first 2 hours after the evening hydrocortisone dose to 3.12 per hour 4 to 7 hours after the dose, corresponding with washout of the evening hydrocortisone dose. Parents of 3- to 10-year-olds reported significantly worse sleep onset delay and decreased sleep duration than both the community and clinical samples, and significantly more night awakenings than the community sample.

**Conclusion:**

Our findings suggest that sleep health is impaired in children with CAH and is an important consideration for both clinical practice and future research.

Congenital adrenal hyperplasia (CAH) due to 21-hydroxylase deficiency (21OHD) is characterized by impaired cortisol synthesis and adrenal androgen overproduction with a wide spectrum of clinical phenotypes based on the severity of the enzymatic defect. CAH is classified as either classic (severe phenotype encompassing salt-wasting and simple-virilizing forms) or nonclassic (NC; mild phenotype). Lifelong cortisol replacement is needed in the classic forms and in symptomatic NC CAH patients. In growing children, short-acting hydrocortisone (HC) is recommended at 10 to 15 mg/m^2^/day in 3 divided doses to avoid the adverse impact on growth from long-acting steroids (1).

Cortisol secretion follows a circadian and ultradian pattern that parallels the daily rhythms of corticotropin-releasing hormone (CRH), which is secreted by the paraventricular nucleus, and adrenocorticotropic hormone (ACTH), highest in the early morning hours and reaching a nadir around midnight (2). The hypothalamic-pituitary-adrenal (HPA) axis activity and circadian rhythmicity of the HPA hormones including cortisol, ACTH, and CRH, play an important role in sleep onset and offset, duration, and distribution of sleep stages across the night (3-5). The relationship between the HPA axis and sleep is complex and bidirectional (6).

In normal physiology, both sleep initiation and slow wave sleep or deep sleep, which are part of non–rapid eye movement (NREM) sleep, coincide with a low HPA-axis activity and low cortisol, CRH, and ACTH levels during the first half of the night (7, 8). On the other hand, rapid eye movement (REM) sleep, awakenings, and sleep offset coincide with increased HPA activity and rising cortisol, CRH, and ACTH levels during the second half of the night in the early morning hours (9). Elevated CRH has been shown to decrease slow wave sleep and sleep duration and to increase light sleep and wakefulness after falling asleep (10). Similar to the HPA axis, normal sleep architecture is also characterized by an ultradian pattern of alternating and repeating cycles of REM and NREM sleep, with each sleep cycle lasting approximately 90 to 120 minutes (11).

Current glucocorticoid replacement therapy in children with CAH does not mimic the natural circadian and ultradian rhythms of cortisol secretion. Hydrocortisone has a short half-life, (median elimination half-life in CAH children of 58 minutes, range 41-105 minutes) (12, 13), allowing most of the HC dose to be eliminated from the body within 4 to 5 hours. As a result, children with CAH are exposed to alternating states of hyper- and hypocortisolemia with low cortisol concentrations and rebounding androgen levels between doses and during the second half of the night when cortisol from the evening HC dose washes out (13, 14), which can lead to HPA hyperactivity and sleep fragmentation. This is consistent with studies in adults with primary adrenal insufficiency, which found poor sleep health using polysomnography (15) and validated questionnaire (16).

Literature on sleep health in children with CAH is sparse (17-20). In previous reports, sleep health was a secondary focus to disease control, comparing dosing regimens, or overall quality of life. To address this knowledge gap, we performed the first study examining both objective and subjective measures of sleep health in children with CAH using wrist actigraphy and validated sleep questionnaires.

## Methods

Participants aged 3 to 18 years, with a diagnosis of 21OHD confirmed by biochemical or molecular testing per chart review, were recruited at the University of Minnesota multidisciplinary CAH clinic. Patients with other forms of adrenal insufficiency were excluded, as were children with previously diagnosed sleep disorders. The study was approved by the University of Minnesota institutional review board. Written informed consent was provided by parent(s) or legal guardian(s) with assent by participants as applicable.

### Subjective Sleep Health Measures

For children 3 to 10 years old, the parents filled out the Children's Sleep Habit Questionnaire (CSHQ), a 33-item validated questionnaire querying children's sleep behavior in 8 domains: bedtime resistance, sleep onset delay, sleep duration, sleep anxiety, night awakenings, parasomnias, sleep disordered breathing, and daytime sleepiness (21-23). The CSHQ has been found to differentiate children with sleep disorders from same-age children with healthy sleep, and it has been used to screen for sleep disorder symptoms in children without CAH (21). A total score of all CSHQ questions in the 8 domains can range from 33 to 99, with higher scores suggesting greater sleep difficulties. A score above 41 indicates a pediatric sleep disorder (0.80 sensitivity and 0.72 specificity) (21). For the CSHQ analysis, our data were compared to community (n = 469) and clinical (n = 154) samples of children 4 to 10 years old reported by Owens et al (21). The community sample included children attending public elementary schools. The clinical sample included children diagnosed with a sleep disorder (behavioral, parasomnia, sleep disordered breathing, etc.) in a pediatric sleep clinic.

Children 8 to 12 and adolescents 13 to 18 years old filled out the Children's Report of Sleep Patterns (CRSP), a self-report validated questionnaire that examines 3 constructs: Sleep Patterns, Sleep Hygiene Index, and Sleep Disturbance Scales (24, 25). Sleep Patterns includes questions about bed and wake times, sleep onset latency, night waking frequency and duration, daytime naps, sleep schedule variability, and subjective sleep quality. The Sleep Hygiene Index includes questions about caffeine use, activities in the hour before bed, sleep location (where the child falls asleep and wakes up), and electronics used at the time of sleep onset. The Sleep Disturbance Scale has questions about bedtime fears/worries, restless legs syndrome symptoms, parasomnias, and insomnia (24, 25). Higher scores represent greater difficulty in that domain.

For the CRSP self-reported surveys, children with CAH were compared to Meltzer's community (n = 278) and clinical (n = 170) samples for 8 to 12 years old, and community (n = 272) and clinical (n = 268) samples for 13 to 18 years old (24, 25). Participants from both age groups were recruited from pediatricians' offices, sleep clinics/laboratories, children's hospitals, schools, and the general population.

### Objective Sleep Health Measures

Children's sleep was objectively measured using the ActiGraph GT3X+ accelerometer (ActiGraph, Pensacola, FL) worn on the nondominant wrist for 1 week. The device uses a solid-state tri-axial accelerometer to collect movement data. An accelerometer is a small device worn on the wrist that detects movement and is used to estimate sleep patterns. By tracking movement data, the device can determine periods of rest and activity, which allows researchers to infer when a person is asleep or awake. It is a commonly used tool in sleep research as it offers an objective method for assessing sleep duration, efficiency, and disturbances, which are key variables when studying conditions like CAH that might impact sleep quality. The accelerometer sampling rate was set as 30 Hz. Parents and children maintained a concurrent daily sleep log to facilitate determining the in-bed and out-of-bed times. Following wear, accelerometer data were downloaded to the ActiLife software (ActiGraph, Pensacola, FL) with 60-second epoch length for processing (26). The Sadeh algorithm was used to score sleep and wake; it has good validity and reliability in children and adolescents (27). Sleep outcomes included: bedtime, waketime, time in bed (TIB), total sleep time (TST), sleep onset latency, minutes of wake after sleep onset, number of awakenings, and sleep efficiency (100 × TST/TIB), averaged across the week (28). Normative objective sleep health data were obtained from a meta-analysis of healthy children (28) and from sleep duration recommendations from the American Academy of Sleep Medicine (29) for comparison.

### Health Variables

Bone age z-scores from chart review were used to assess the cumulative exposure of bones to estrogen, primarily through aromatization of adrenal androgen to estrogen. Bone age is an integrated but lagging indicator of antecedent control, which complements clinical signs and symptoms in between clinic visits (14). Age- and sex-adjusted bone age z-scores were calculated using reference values from Greulich and Pyle (30). Additional values obtained from chart review include body mass index (BMI) z-score, hydrocortisone regimen, child age, sex, puberty status, and height.

### Statistical Methods

Descriptive statistics for all sleep health variables are presented as mean and SD. Differences between subjective sleep health variables for children with CAH and the community and clinical samples previously reported in the literature (21, 24, 25) were assessed with 2-sample *t* tests. Equal-variance *t* tests were reported except when the unequal-variance test gave a qualitative different result, in which case the latter is reported as it is more conservative. Objective sleep health variables for children with CAH were compared to sleep duration recommendations (29) and normative data in healthy children (28) for sleep onset latency, sleep efficiency, and minutes of wake after sleep onset using 2-sample *t* tests, with standard errors computed as the width of the 95% CI divided by 3.92. Finally, associations between bone age z-scores, BMI z-scores, and evening hydrocortisone dose with objective sleep health variables were examined with multiple linear regression, adjusting for age, sex, puberty status, and height (for BMI z-score). Adjusted associations of sleep outcomes with child's average time of last dose were estimated and testing using multiple linear regression.

All analyses were done using JMP (v. 16.1.0 Pro, SAS Institute Inc., Cary NC USA).

## Results

**Figure 1.**
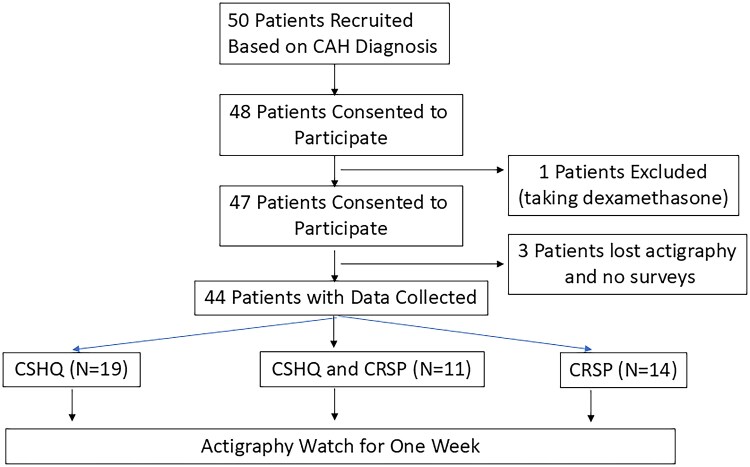
Consort diagram of participants in the study. All participants had data collected via surveys and actigraphy. The CSHQ was provided for parents of children 3 to 10 years old and 1 child 12 years old and the CRSP was provided for anyone over 8 years old. Given the age overlap, some individuals had both a CSHQ and CRSP survey completed. As CSHQ has been used in children older than 10 years in other studies, the data from one 12-year-old was included in the analysis (40).

A total of 47 patients with classic CAH (25 salt-wasting, 23 male) were enrolled in the study (see Fig. 1 and Table 1), of whom 44 patients had full data collection (3 were missing actigraphy data). Thirty parents filled out the CSHQ: 19 participants only had a parent fill out the CSHQ survey while 11 had both the parental CSHQ and self-reported CRSP. Fourteen children completed only a self-reported CRSP.

**Table 1.** Participant characteristics

Characteristic	Mean (SD)	Range
Diagnosis, SW, n (%)	25 (57%)	NA
Age at CAH diagnosis (years)*^a^*	1.2 (2.4)	0.01 to 10
Age at study participation (years)	8.5 (3.4)	3 to 15
Sex, male, n (%)	23 (52%)	NA
Puberty (Tanner stage ≥2), n (%)	9 (20%)	NA
Height Z-score	0.82 (1.25)	−1.56 to 4.62
BMI Z-Score	0.83 (0.96)	−1.19 to 2.62
Bone age Z-score	1.88 (2.11)	−2.57 to 6.02
Total daily HC dose, mg/m^2^/day	11.3 (2.96)	5.2 to 16.9
Bedtime HC dose, mg/m^2^/day	2.15 (0.77)	0.78 to 3.62
Mean time of bedtime dose	8:48 Pm (67 minutes)	7:30 to 11:00 Pm
First morning HC dose, mg/m^2^/day	5.3 (2.7)	1.00 to 12.5
Mean time of first morning dose	5:48 Am (53 minutes)	5:00 to 6:00 Am
Second morning HC dose, mg/m^2^/day*^b^*	2.9 (1.6)	0.9 to 7.5

N = 44 except as otherwise noted. Data presented as average (SD) unless otherwise stated.

Abbreviations: BMI, body mass index; CAH, congenital adrenal hyperplasia; HC, hydrocortisone; SW, salt-wasting.

^*a*^N = 43.

^*b*^Patients who split their morning dose were instructed to take the second dose 2 hours after the first. The exact time of the second dose was not recorded.

Children were on 3 to 5 doses of hydrocortisone a day following a circadian dose distribution (average total daily dose 11.3 [SD 2.96] mg/m^2^/days) with 55.5% of the dose in the morning, 25.5% in the afternoon and 19% in the evening. The average reported bedtime dose was 2.15 (0.77) mg/m^2^/days taken at an average time of 8:48 Pm (SD 67 minutes) and the average first morning dose was 5.3 (SD 2.7) mg/m^2^/days taken at an average time of 5:49 Am (SD 53 minutes) (Table 1). None of the children or their parents reported polyuria or nocturia. None of the participants had low sodium or elevated potassium or salt-wasting crisis during the study period and the preceding year. Mean plasma renin activity at entry of study was 4.93 ng/mL/h (SD 3.22), indicating that these patients did not have salt-wasting.

### Subjective Sleep Health

#### Parent-reported

The parents of children with CAH reported significantly (*P* < .05) worse scores compared to Owen's community sample (21) for bedtime resistance, sleep onset delay, sleep duration, sleep anxiety, night wakening, parasomnias, and daytime sleepiness (Table 2). The CAH group also had significantly worse scores than children with sleep disorders for bedtime resistance, sleep onset delay, and sleep duration. Children with CAH did not differ significantly from the community or clinical sample in the disordered breathing subscale.

**Table 2.** CSHQ subscale items reported by parents of 3- to 10-year-olds with CAH compared to community and clinical normative samples

CSHQ sleep domain	CAH sample n = 30	Community sample n = 469	Clinical sample n = 154
Bedtime resistance	11.39 (2.11)	7.06 (1.89)*^a^*	9.43 (3.49)*^a^*
Sleep onset delay	2.63 (0.61)	1.25 (0.53)*^a^*	1.80 (0.88)*^a^*
Sleep duration	6.97 (0.74)	3.41 (0.93)*^a^*	4.94 (1.98)*^a^*
Sleep anxiety	6.10 (2.4)	4.89 (1.45)*^a^*	7.09 (2.44)
Night wakening	4.67 (2.04)	3.51 (0.89)*^a^*	5.69 (1.6)*^a^*
Parasomnias	9.72 (2.34)	8.11 (1.25)*^a^*	11.22 (2.53)*^a^*
Disordered breathing	3.24 (0.64)	3.24 (0.63)	4.71 (2.54)
Daytime sleepiness	12.33 (2.22)	9.64 (2.8)*^a^*	11.99 (3.39)

All values are mean (SD).

Abbreviations: CAH, congenital adrenal hyperplasia; CSHQ, Child Sleep Habits Questionnaire.

^*a*^Significant difference from CAH sample in 2-sample *t* test, *P* < .05.

#### Child (self)-reported

Children 8 to 12 years with CAH reported significantly better scores on the CRSP Sleep Hygiene Scale compared to the community and clinical samples for caffeine intake, activities before bed, and sleep location (Table 3). Children with CAH did not differ significantly from the community or clinical sample for electronic use at bedtime, restless legs, insomnia, or parasomnias. Children with CAH had significantly better scores compared to children with sleep disorders for bedtime fears/worries but did not differ significantly compared to the community sample.

**Table 3.** CRSP Sleep Hygiene Scale items for participants with CAH compared to community and clinical samples

Sleep Hygiene Scale ages 8 to 12 years sleep domain	CAH sample n = 18	Community sample n = 278	Clinical sample n = 170
Caffeine	4.39 (1.65)	6.05 (2.3)*^a^*	6.48 (2.4)*^a^*
Activities before bed	13.67 (3.56)	16.45 (3.1)*^a^*	17.01(3.2)*^a^*
Sleep location	7.28 (2.14)	9.73 (4.2)*^a^*	11.66 (4.5)*^a^*
Electronic use bedtime	5.61 (2.43)	5.60 (2.5)	6.82 (3.2)
Bedtime fears/worries	3.00 (1.64)	3.44 (1.7)	3.95 (2.1)*^a,b^*
Restless legs	8.33 (2.09)	9.33 (3.1)	10.28 (0.28)
Insomnias	11.67 (4.43)	10.91 (3.7)	12.72 (4.6)
Parasomnias	2.72 (0.96)	2.62 (0.9)	3.19 (1.2)

Sleep Hygiene Scale ages 13 to 18 years sleep domain	CAH sample n = 7	Community sample n = 272	Clinical sample n = 268
Caffeine	5.86 (1.86)	7.09 (2.5)	7.24 (2.36)
Activities before bed	19.71 (4.19)	17.39 (2.96)	18.04 (3.03)
Sleep location	8.86 (4.18)	2.80*^a^* (0.53)*^a^*	3.04 (0.64)*^a^*
Electronic use bedtime	6.13 (3.36)	5.76 (2.58)	6.69 (2.96)
Bedtime fears/worries	5.00 (1.53)	3.50 (1.49)*^a^*	3.41 (1.74)*^a^*
Restless legs	8.29 (2.63)	4.35 (1.38)*^a^*	4.00 (1.23)*^a^*
Insomnias	8.00 (2.45)	9.77 (3.47)	9.39 (3.32)
Parasomnias	2.43 (0.79)	4.02 (1.5)*^a^*	3.72 (1.5)*^a^*

All values are mean (SD).

Abbreviations: CAH, congenital adrenal hyperplasia; CRSP, Children's Report of Sleep Patterns.

^*a*^Significant difference from the CAH sample on a 2-sample *t* test, *P* < .05. The analysis assumes equal variance except, *^b^*where the unequal variance analysis is reported, as described in the statistical methods section.

For the older age group, 13 to 18 years old, the CAH sample had significantly worse scores in the CRSP Sleep Hygiene Scale compared to the community and clinical samples for sleep location, bedtime fears/worries, and restless legs (Table 3). For the domains of caffeine intake, activities before bed, electronic use at bedtime, and insomnia, the CAH group did not differ significantly from the community or clinical sample. The CAH group reported significantly better scores than both the community and clinical sample for the parasomnia sleep domain.

In the CRSP sleep health descriptive characteristics (Supplementary Table S1) (31), 84% of children 8 to 12 years old and 75% of those 13 to 18 years old reported that they were good/great sleepers; 79% of children 8 to 12 years old and 100% of those 13 to 18 years old reported that they get the right amount of sleep.

### Objective Sleep Health

In the sample of children with CAH, average total sleep time was 7.56 hours per night. This is less than the recommended minimum number of hours of sleep for all ages based on the American Academy of Sleep Medicine (29) (Table 4). The average sleep latency for the CAH group was 24.9 minutes which did not differ significantly from an average of 19.4 minutes of healthy children (28). Sleep efficiency for children with CAH was significantly worse than the pooled mean estimates of healthy children at 75.7 or 78.8 minutes compared to 86.3 and 88.3 minutes, respectively (Table 5). Also, the wake after sleep onset time in minutes was significantly longer at 122.5 minutes for children with CAH compared to 55 minutes for healthy children in the meta-analysis (28).

**Table 4.** Actigraphy-derived sleep duration of CAH patients compared to American Academy of Sleep Medicine recommendations

Age	CAH patients *Hours (SD)*	AASM guidelines *Hours*
3 to 5 years	7.75 (0.93) n = 12	10 to 13
6 to 12 years	7.68 (0.75) n = 25	9 to 12
13 to 18 years	6.89 (0.5) n = 7	8 to 10

Abbreviations: AASM, American Academy of Sleep Medicine; CAH, congenital adrenal hyperplasia.

**Table 5.** Actigraphy-derived sleep variables for children with CAH compared to a healthy control sample

Actigraphy sleep variable	CAH average (SD), n = 44	Average of healthy controls (95% CI)
Average bedtime	9:10 Pm (69 minutes)	NA
Average waketime	7:17 Am (56 minutes)	NA
Sleep onset latency (min)	24.9 (18.1)	19.4 (16.6, 22.1)
Sleep efficiency (%)	75.7 (7.2)	86.3 (84.4, 88.2)*^a^*
Wake after sleep onset (min)	122.5 (45.8)	55 (43, 68)*^a^*

Abbreviation: CAH, congenital adrenal hyperplasia.

^*a*^Significant difference from CAH sample using the 2-sample *t* test, *P* < .05.

The average number of awakenings per hour after each participant fell asleep (Table 6) showed a trend toward an increase starting 4 hours after the evening hydrocortisone dose. There was no significant correlation between the average time of last hydrocortisone dose (eg, 7 Pm vs 8 Pm vs 9 Pm vs 10 Pm) and average sleep efficiency or wake time after sleep onset for both unadjusted and adjusted for age and puberty analyses. Bone age z-scores (measure of disease control), BMI z-scores, and evening hydrocortisone dose had small and nonsignificant (*P* > .05) associations with objective sleep health variables after adjusting for age, sex, puberty status, and height (for BMI z-scores).

**Table 6.** After sleep onset, the average number of actigraphy-estimated awakenings per hour following evening hydrocortisone dose for participants with CAH

Hours after HC evening dose	Number of patients asleep per hour post HC dose	Average number of awakenings per hour	Standard error
0 to 1	38	1.43	0.2
1 to 2	42	1.90	0.19
2 to 3	43	2.26	0.19
3 to 4	43	3.02	0.19
4 to 5	44	2.95	0.19
5 to 6	44	3.21	0.19
6 to 7	44	3.20	0.19
7 to 8	43	3.12	0.19
8 to 9	36	2.57	0.21
9 to 10	22	2.73	0.26
10 to 11	14	2.22	0.33
11 to 12	6	3.9	0.51
12 to 13	2	3.0	0.88

Abbreviations: CAH, congenital adrenal hyperplasia; HC, hydrocortisone.

## Discussion

The current study reported on objective and subjective sleep health using actigraphy and validated sleep questionnaires in a sample of children with CAH. Measured by actigraphy, children with CAH obtained less than the recommended number of hours of sleep for age (29) and had more awakenings and worse sleep efficiency compared to previously reported healthy children (28). Similarly, parents of 3- to 10-year-old children with CAH reported significantly decreased sleep duration and more night awakenings compared to a community sample (21). Parents also perceived that children with CAH had more bedtime resistance, anxiety, and daytime sleepiness compared to the community sample. In contrast to the actigraphy findings of decreased sleep duration, increased awake time and poor sleep efficiency, > 75% of 8- to 18-year-olds reported that they were good/great sleepers and > 79% reported that they consistently got the right amount of sleep in the CRSP sleep health descriptive characteristics (Supplementary Table S1). These findings underscore that subjective perception of sleep may not match objective sleep measurement but that both are important to evaluate in children with CAH.

Only 2 previous studies of children with CAH reported on sleep health (19, 20). A 6-week prospective crossover study of 37 children with CAH (age range, 4-17 years) found no difference in sleep as assessed with a single general rating (score between 0 and 5) reported daily by children with CAH and their parents for circadian vs reverse circadian HC dosing (higher dose in the morning vs higher dose in the evening) (20). A 4-week open-label, randomized crossover study of 15 children with classic CAH (age range, 6-17 years) objectively estimated sleep using actigraphy during 2 weeks of circadian vs 2 weeks of reverse circadian HC dosing (19). The authors reported no significant differences in sleep, including duration, efficiency, or latency, by HC dosing regimen. However, total awake time spent after sleep onset was not reported and true sleep time was not broken down by age, as optimal true sleep time varies by age (29). Notably, these studies did not compare sleep in children with CAH to that in control samples.

Consistent with hydrocortisone's short half-life, the number of awakenings trended toward increasing starting 4 hours after the evening hydrocortisone dose, corresponding with the time that cortisol pharmacokinetic studies have shown cortisol concentrations are closer to pre-dose concentrations (12, 13). Loss of cortisol's inhibitory feedback on the HPA axis would lead to subsequent increase in CRH levels that most likely would contribute to wakefulness (12, 32, 33). The effect of low cortisol and subsequent elevations of ACTH and CRH on sleep architecture were demonstrated in 10 adult patients with primary adrenal insufficiency, whose polysomnography studies showed increased wake after sleep onset and fragmented sleep after withholding of glucocorticoid medication for 1.5 days (15).

Although much of the previous literature has emphasized the role of cortisol in sleep, cortisol may not be the primary cause of the sleep disturbance but rather may be a marker of CRH activity (3). CRH has been proposed as the common cause impacting HPA axis activity and sleep, with glucocorticoid having an indirect role through CRH regulation (33). High CRH and cortisol levels at bedtime in patients with Cushing disease has been associated with longer sleep latency, decreased time spent in deep sleep, and increased time spent in REM and awake (34, 35). A similar effect is seen during sleep deprivation, where hyperactivity of the HPA axis and sympathetic nervous system can lead to high CRH, cortisol, and norepinephrine levels, resulting in sleep fragmentation, decreased time spent in deep sleep, shortened sleep duration, and daytime dysfunction (36). However, this has not been previously examined in children with CAH.

In our study, sleep onset latency was not significantly different in children with CAH compared with a healthy normative population. All participants in the study were on a circadian hydrocortisone regimen (higher hydrocortisone dose in the morning and lower in the evening). A hydrocortisone dose–related effect has been shown in healthy male subjects, in which lower hydrocortisone doses can increase deep sleep, while at higher doses it can increase sleep latency and wakefulness (37). It has been proposed that low cortisol levels are associated with mineralocorticoid receptor binding in the hippocampus, which mediates a negative feedback on hypothalamic CRH secretion (3). In contrast, at higher cortisol levels, amygdala glucocorticoid receptors may be activated, which exerts excitatory feedback on CRH, increasing CRH secretion and therefore promoting sleep disruption (3).

Limitations to our study are those arising from using questionnaires, which are subject to self-reporting and recall bias (38). Also, our study could not use polysomnography, which is the gold standard for sleep assessment, so sleep architecture was not assessed. While actigraphy offers high quality objective assessment of sleep over longer periods in the home environment, it can overestimate sleep if there is minimal movement while awake in bed or it can underestimate sleep if there is excess movement during sleep. Another limitation is that this study did not assess or control for mental health diagnoses, such as anxiety, that can impact sleep. It has been reported that children with CAH have higher anxiety than healthy controls (39); further research is needed to explore this association with sleep health. Also, while we did not find a significant association between evening hydrocortisone dose and disease control as reflected by the bone age z-scores, a larger scale study is needed to further evaluate the link of these variables to sleep health.

In conclusion, our findings suggest that sleep health may be impaired in children with CAH and is an important consideration for both clinical practice and future research. Further studies in children with CAH are needed to quantitatively evaluate other aspects of sleep health, such as sleep architecture, with cortisol exposures resulting from various glucocorticoid regimens.

## Data Availability

Restrictions apply to the availability of data generated or analyzed during this study to preserve patient confidentiality. The corresponding author will, on request, detail the restrictions and any conditions under which access to some data may be provided.

## Abbreviations

21OHD: 21-hydroxylase deficiency
ACTH: adrenocorticotropic hormone
BMI: body mass index
CAH: congenital adrenal hyperplasia
CRH: corticotropin-releasing hormone
CRSP: Children’s Report of Sleep Patterns
CSHQ: Children's Sleep Habit Questionnaire
HC: hydrocortisone
HPA: hypothalamic-pituitary-adrenal
NC: nonclassic
NREM: non–rapid eye movement
REM: rapid eye movement
